# Differential Effects of Trp53 Alterations in Murine Colorectal Cancer

**DOI:** 10.3390/cancers13040808

**Published:** 2021-02-15

**Authors:** Alexander M. Betzler, Lahiri K. Nanduri, Barbara Hissa, Linda Blickensdörfer, Michael H. Muders, Janine Roy, Moritz Jesinghaus, Katja Steiger, Wilko Weichert, Matthias Kloor, Barbara Klink, Michael Schroeder, Massimiliano Mazzone, Jürgen Weitz, Christoph Reissfelder, Nuh N. Rahbari, Sebastian Schölch

**Affiliations:** 1 Department of Surgery, Universitätsmedizin Mannheim, Medical Faculty Mannheim, Heidelberg University, 68167 Mannheim, Germany; a.betzler@umm.de (A.M.B.); bhissa@beilstein-institut.de (B.H.); christoph.reissfelder@umm.de (C.R.); 2 Department of Gastrointestinal, Thoracic and Vascular Surgery, Medizinische Fakultät Carl Gustav Carus, Technische Universität Dresden, 01307 Dresden, Germany; lahirin@flowcell.co (L.K.N.); juergen.weitz@ukdd.de (J.W.); 3 Department of General, Gastrointestinal and Transplant Surgery, Ruprecht-Karls-Universität Heidelberg, 69120 Heidelberg, Germany; linda4@gmx.net; 4 Institute of Pathology, University of Bonn Medical Center, 53127 Bonn, Germany; Michael.Muders@ukbonn.de; 5 Department of Bioinformatics, Biotechnology Center, Technische Universität Dresden, 01307 Dresden, Germany; janine.roy@biotec.tu-dresden.de (J.R.); michael.schroeder@tu-dresden.de (M.S.); 6 Institute of Pathology, Technische Universität München, 81675 München, Germany; moritz.jesinghaus@tum.de (M.J.); katja.steiger@tum.de (K.S.); wilko.weichert@tum.de (W.W.); 7 Department of Applied Tumor Biology, Institute of Pathology, Ruprecht-Karls-Universität Heidelberg, 69120 Heidelberg, Germany; matthias.kloor@med.uni-heidelberg.de; 8 Clinical Cooperation Unit Applied Tumor Biology, German Cancer Research Center (DKFZ), 69120 Heidelberg, Germany; 9 Institute of Clinical Genetics, Medizinische Fakultät Carl Gustav Carus, Technische Universität Dresden, 01307 Dresden, Germany; barbara.klink@ukdd.de; 10 Laboratory of Tumor Inflammation and Angiogenesis, Center for Cancer Biology (CCB), VIB, 3000 Leuven, Belgium; massimiliano.mazzone@kuleuven.vib.be; 11 Laboratory of Tumor Inflammation and Angiogenesis, Department of Oncology, KU Leuven, 3000 Leuven, Belgium; 12 Junior Clinical Cooperation Unit Translational Surgical Oncology (A430), German Cancer Research Center (DKFZ), 69120 Heidelberg, Germany

**Keywords:** colorectal cancer, genetically engineered mouse model, GEMM, adenovirus, Apc, Kras, Trp53, TP53, metastasis, preclinical studies

## Abstract

**Simple Summary:**

Although colorectal cancer is among the most frequent malignant tumors, there are currently no mouse models available that reliably mimic both tumor biology as well as treatment response. In this article, we describe a novel mouse model in which mutations relevant to colorectal cancer are induced in mice, leading to tumor formation in the distal colon. The tumors are monitored via colonoscopy, and the survival and the histology of the tumors are examined. We demonstrate that this model can closely model the human disease clinically, histologically and genetically. In addition, the response of this model to classical colorectal cancer treatments is more realistic than that of other mouse models. The effects of different mutations in the Trp53 gene on tumor cells show striking differences, similar to the effects in other tumor diseases. In summary, the new model allows more accurate and predictive experiments in colorectal cancer.

**Abstract:**

Background: Colorectal cancer (CRC) development is a multi-step process resulting in the accumulation of genetic alterations. Despite its high incidence, there are currently no mouse models that accurately recapitulate this process and mimic sporadic CRC. We aimed to develop and characterize a genetically engineered mouse model (GEMM) of Apc/Kras/Trp53 mutant CRC, the most frequent genetic subtype of CRC. Methods: Tumors were induced in mice with conditional mutations or knockouts in Apc, Kras, and Trp53 by a segmental adeno-cre viral infection, monitored via colonoscopy and characterized on multiple levels via immunohistochemistry and next-generation sequencing. Results: The model accurately recapitulates human colorectal carcinogenesis clinically, histologically and genetically. The Trp53 R172H hotspot mutation leads to significantly increased metastatic capacity. The effects of Trp53 alterations, as well as the response to treatment of this model, are similar to human CRC. Exome sequencing revealed spontaneous protein-modifying alterations in multiple CRC-related genes and oncogenic pathways, resulting in a genetic landscape resembling human CRC. Conclusions: This model realistically mimics human CRC in many aspects, allows new insights into the role of TP53 in CRC, enables highly predictive preclinical studies and demonstrates the value of GEMMs in current translational cancer research and drug development.

## 1. Introduction

Colorectal cancer (CRC) is genetically a highly heterogeneous disease [1]. Canonical CRC evolution starts with mutations in the *APC* gene [2], often followed by oncogenic *KRAS* and *TP53* mutations [3]. Among numerous attempts to classify CRC, the colon cancer subtype (CCS) system uses genetic, epigenetic, and clinical features [4]. It describes three subtypes, among which CCS1 is the most frequent (49% of CRC cases), featuring simultaneous mutations in *APC*, *KRAS,* and *TP53* [5].

Most currently used CRC mouse models are based on the subcutaneous or orthotopic injection of cell lines into immunodeficient or, in the case of murine cell lines, immunocompetent mice [6,7,8,9]. This results in anaplastic tumors that typically respond well to cytotoxic therapy and may overestimate the efficacy of novel compounds [10]. This poor predictive value of current CRC models contributes to the high clinical failure rate of novel compounds despite proven preclinical activity [11]. In order to overcome this issue, genetically engineered mouse models (GEMMs) have been developed for numerous tumor entities over the past few years [11,12]. In GEMMs, the genetic hallmarks of human disease are reproduced in a tissue-specific manner. This leads to genuine mouse tumors that often recapitulate human disease with astonishing accuracy [13,14,15,16,17]. The only currently known GEMM of sporadic (i.e., unifocal) CRC employs segmental cre-recombination in the colon via surgical clamping and infection with adeno-cre virus [18,19,20]. In this model, mice with conditional mutations of *Apc* and *Kras* and other oncogenes are segmentally infected and subsequently develop one or few adenomas of the distal colon, eventually invasive carcinoma and rarely metastatic disease.

Despite the high incidence of the CCS1 subtype, no such GEMM is currently available to mimic its genetic complexity. In addition, although most *Trp53* (the murine counterpart of human TP53) alterations involve mutations rather than complete *Trp53* loss, no CRC GEMM of mutant *Trp53* has been described yet.

We here present and comprehensively characterize a novel GEMM of compound *Apc*/*Kras*/*Trp53* mutated CRC (CCS1) that enables valid research of the biology and treatment response of the most common human CRC subtype. Using this mouse model, we evaluated the differential effects of various genetic alterations in the Trp53 gene in CTC.

## 2. Materials and Methods

### 2.1. Cell Culture

The human CRC cell line HCT116 (CCL-247, ATCC, Manassas, VA, USA) was propagated in DMEM (supplemented with 10% heat-inactivated fetal bovine serum, 100 U/mL penicillin, and 100 g/mL streptomycin (all from GE Healthcare, Rimbach, Germany). The cell line was regularly tested for contamination and authenticated via single nucleotide polymorphism (SNP) profiling by an external service provider (Multiplexion, Heidelberg, Germany).

### 2.2. Mice

The mouse lines Apc^fl/fl^ (B6.Cg-*Apc^tm2Rak^*/Nci [21]), Kras^LSL-G12D^ (B6.129-*Kras^tm4Tyj^*/Nci [22]), Trp53^LSL-R172H^ (129S4-*Trp53^tm2Tyj^*/Nci [23]), and Trp53^fl/fl^ (FVB.129P2-*Trp53^tm1Brn^*/Nci [24]) were generously provided by the NCI Mouse Repository (Frederick, MD, USA). Trp53^LSL-R172H^ and Trp53^fl/fl^ mice were backcrossed to C57Bl/6N mice (Charles River Laboratories, Sulzfeld, Germany) for >10 generations to transfer the p53 alleles to a C57Bl/6N background. Thus, all mice described in this paper are on C57Bl/6N genetic background. The mice were then crossed to obtain the following genotypes (the acronyms in parentheses are used henceforth):

i. *Apc*^fl/fl^ (“Apc”);
ii. *Apc*^fl/fl^/*Kras*^LSL-G12D/+^ (“AK”);
iii. *Apc*^fl/fl^/*Kras*^LSL-G12D/+^ /*Trp53*^fl/fl^ (“AKPfl”);
iv. *Apc*^fl/fl^/*Kras*^LSL-G12D/+^ /*Trp53*^LSL-R172H/+^ (“AKPr”);

Genotyping was performed on tail biopsies by TransnetYX (Memphis, TN, USA).

### 2.3. Animal Experiments

All animal experiments were approved by Regierungspräsidium Karlsruhe and Landesdirektion Sachsen prior to initiation of the experiments (AZ G-188/11). Mice were housed in a specific pathogen-free environment with a 12 h light/dark cycle and were given *ad libitum* access to standard laboratory diet and water.

For detailed descriptions of surgical tumor induction, colonoscopy, and treatment protocols, please see supplemental methods. Details about the HCT116 subcutaneous (s.c.) injection experiments can also be found in the supplementary methods in the supplementary file.

### 2.4. Migration Assay

Please see supplementary methods in the supplementary file.

### 2.5. Histology

Depending on the experimental group, mice were either euthanized on pre-defined time points or when under distress. Dissection was performed, and colon, liver and lung were harvested. Snap frozen, formalin-fixed and paraffin-embedded (FFPE) tissue specimens were processed using standard laboratory methods. Further details about histology protocols can be found in the supplementary methods in the supplementary file.

Pathologic assessment was performed according to current consensus papers about mouse CRC pathology; for details, please see supplementary methods in the supplementary file.

### 2.6. Immunohistochemistry

Immunohistochemistry was performed on a fully automated immunohistochemistry Leica BondMax RXm system (Leica, Wetzlar, Germany). For details, please see supplementary methods in the supplementary file.

### 2.7. Microsatellite Instability Testing

Please see supplementary methods in the supplementary file.

### 2.8. Next Generation Sequencing and Data Analysis

Please see supplementary methods in the supplementary file.

### 2.9. Statistics

Statistical analysis was done by the χ^2^ test for categorical data and the log-rank test for survival analyses. Survival was depicted using the Kaplan-Meier method. For multiple comparisons, ANOVA with Tukey’s multiple comparisons was used. *p* < 0.05 was considered statistically significant, *p* values were adjusted for multiple testing whenever applicable. Graphpad Prism 7 (Graphpad Software Inc., San Diego, CA, USA) was used for statistical analyses and data plotting. Statistically significant values are indicated by asterisks (key: * *p* < 0.05; ** *p* < 0.01; *** *p* < 0.001; **** *p* < 0.0001).

## 3. Results

### 3.1. Sequential Addition of Mutations Accelerates Malignant Tumor Formation

**Figure 1:**
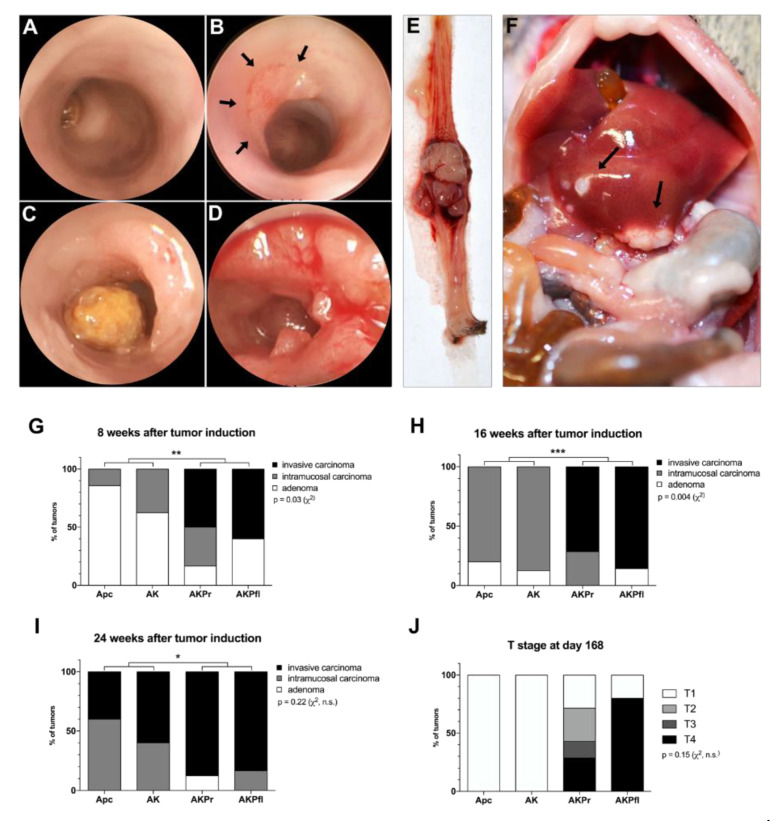
*Trp53* conditional mutations lead to fast tumor progression and metastatic behavior in murine CRC. (**A**–**D**) After tumor induction via adeno-cre viral infection, mice were submitted to colonoscopy. Representative images were collected for (**A**) normal colon prior to adeno-cre infection, (**B**) early adenoma (arrows) in an Apc mouse, (**C**) preinvasive intramucosal carcinoma in an AK mouse, and (**D**) advanced adenocarcinoma in an AKPr mouse (note contact vulnerability). All above diagnoses were made histologically after colonoscopy. (**E**) Distal colon with a large tumor in an AKPfl mouse. (**F**) Liver metastases (arrows) in an AKPr mouse. (**G**–**I)** Quantification of tumor invasiveness at 8 weeks, 16 weeks, and 24 weeks after tumor induction. **(J)** Local T staging of all invasive tumors after 24 weeks of tumor growth. * *p* < 0.05; ** *p* < 0.01; *** *p* < 0.001. Apc, Apc^fl/fl^ mice; AK, Apc^fl/fl^/Kras^LSL-G12D/+^ mice; AKPr, Apc^fl/fl^/Kras^LSL-G12D/+^/Trp53^LSL-R172H/+^ mice; AKPfl, Apc^fl/fl^/Kras^LSL-G12D/+^/Trp53^fl /fl^ mice.

**Figure 2:**
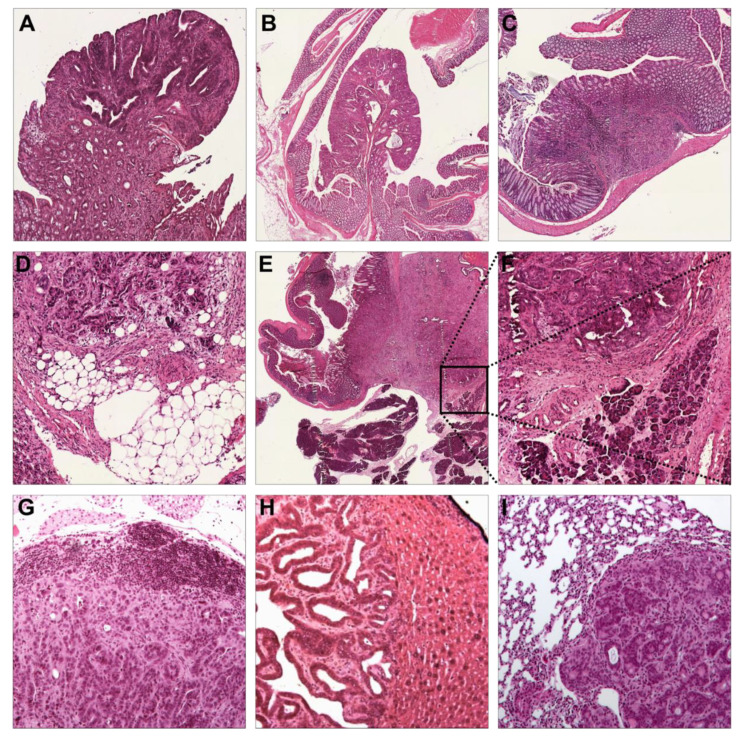
Histopathology analysis confirms the invasiveness and metastatic potential of the mouse model. (**A**). Villous adenoma (Apc, 4×). (**B**) Tubular adenoma (AK, 2×). (**C**) Focally invasive adenocarcinoma invading the submucosa (T1b sm3, AK, 4×). (**D**) Scirrhous adenocarcinoma invading pericolic adipose tissue (T4, AKPfl, 10×). (**E**) Locally advanced, scirrhous adenocarcinoma invading pancreatic acinar tissue (T4, AKPfl, 2×). (**F**) Magnification (10×) of *E*. (**G**) Mesenteric lymph node metastasis (AKPr, 10×). (**H**) Liver metastasis (AKPr, 10×). (**I**) Lung metastasis (AKPr, 10×). Apc, Apc^fl/fl^ mice; AK, Apc^fl/fl^/Kras^LSL-G12D/+^ mice; AKPr, Apc^fl/fl^/Kras^LSL-G12D/+^/Trp53^LSL-R172H/+^ mice; AKPfl, Apc^fl/fl^/Kras^LSL-G12D/+^/Trp53^fl /fl^ mice.

Tumors were surgically induced by segmental colonic infection with adeno-cre virus. Postoperatively, tumor growth was monitored every other week via colonoscopy (Figure 1A–D). At 56, 112, and 168 days after tumor induction, 5–8 mice/genotype/time point were euthanized and the tumors were harvested (Figure 1E) for histopathologic analysis [25,26]. The tumors in the GEMM were almost exclusively adenocarcinomas with typical colorectal adenocarcinoma histomorphology (Figure 2A–I).

In mice without alterations in *Trp53*, histological analysis revealed that most Apc mice had developed villous adenoma and rarely intramucosal carcinoma at 56 days after tumor induction (Figure 1G). The addition of the oncogenic *Kras* G12D mutation (AK mice) accelerated adenoma formation and increased the frequency of intramucosal carcinoma. Alterations in *Trp53* led to the early development of invasive tumors.

After 112 days of tumor induction (Figure 1H), most adenomas in Apc and AK mice had progressed to intramucosal carcinoma. However, no invasive tumors were identified in those groups. Again, *Trp53* alterations led to faster progression, resulting in the majority of *Trp53* mutant (AKPr) or *Trp53* deficient (AKPfl) tumors being classified as invasive adenocarcinomas.

On day 168, all Apc mice were diagnosed with intramucosal or invasive adenocarcinoma (Figure 1I) and AK mice had developed a slightly higher proportion of invasive tumors, demonstrating genetic and clinical tumor progression over time. Mice with alterations in the *Trp53* gene showed significantly more invasive tumors than *Trp53* WT mice.

Most tumors exhibited poor or anaplastic differentiation, especially in *Trp53* mutant mice, in which no well-differentiated tumors were seen (Figure S1).

### 3.2. Immunohistochemistry Confirms Colorectal Origin of Primary Tumors and Metastases

**Figure 3:**
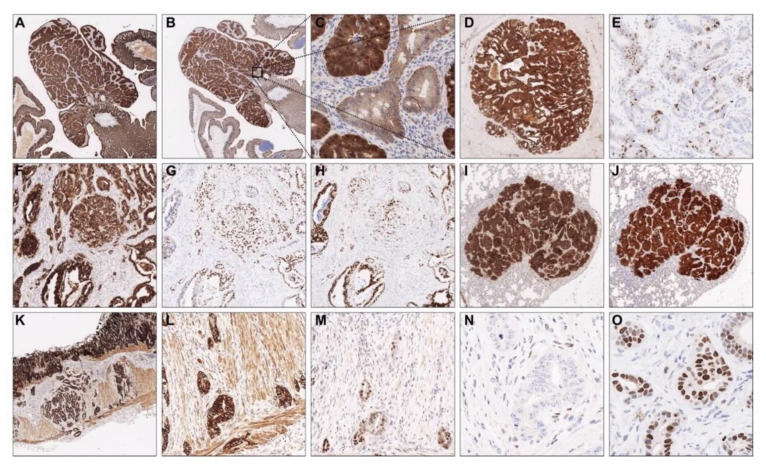
Immunohistochemical analysis confirms the colorectal origin of the tumors and metastases. (**A**–**C**) EPCAM ((**A**), 2×) and β-catenin ((**B**), 2×) expression in a tubular adenoma (Apc). Note the accumulation of β-catenin in neoplastic glands as compared to non-transformed glands ((**C**), 20×). (**D**,**E**) EPCAM ((**D**), 4×) and CDX2 ((**E**), 4×) expression demonstrate the colorectal origin of liver metastasis (AK). (**F**–**H**) Invasive tumor cells in an AKPr mouse: EPCAM ((**F**), 10×), P53 ((**G**), 10×) and CDX2 ((**H**), 10×). (**I**,**J**) EPCAM ((**I**), 4×) and β-catenin ((**J**), 4×) positivity confirm a colorectal lung metastasis (AK). (**K**–**M**) AKPfl tumor penetrating the muscularis propria: EPCAM, ((**K**) (4×), (**L**) (20×)), CDX2 ((**M**), 20×). (**N**,**O**) Loss of P53 expression in AKPfl ((**N**), 40×) and nuclear accumulation of P53 in AKPr ((**O**), 40×) in direct comparison. Apc, Apc^fl/fl^ mice; AK, Apc^fl/fl^/Kras^LSL-G12D/+^ mice; AKPr, Apc^fl/fl^/Kras^LSL-G12D/+^/Trp53^LSL-R172H/+^ mice; AKPfl, Apc^fl/fl^/Kras^LSL-G12D/+^/Trp53^fl /fl^ mice.

In order to further characterize the tumors, we performed immunohistochemical stainings for EpCAM, β-catenin, CDX2 and p53 on selected tumors (Figure 3). All tumors and distant metastases were uniformly and strongly positive for EpCAM (Figure 3A,D,F,I,K,L), confirming their epithelial origin. As expected, the knockout of *Apc* led to strong accumulation of β-catenin in all tumors and metastatic lesions (Figure 3B,C,J). As Apc is pivotal for β-catenin phosphorylation and consecutive proteasomal degradation [27], the accumulation of β-catenin independently confirmed the efficiency of *Apc* loss in the GEMM. Partial nuclear positivity for CDX2 corroborated the colorectal origin of the examined tumors and distant metastases (Figure 3E,H,M) [28]. In AKPfl tumor cells, the expression of p53 protein was entirely lost (Figure 3N), whereas the mutant p53 protein accumulated in the nuclei of AKPr tumor cells (Figure 3G,O), a phenomenon well-known for many mutant forms of p53, including p53 R172H/R175H [29]. Of note, stromal cells in tumors of all genotypes exhibited physiological expression patterns of EpCAM, β-catenin, CDX2 and p53, thus ruling out accidental infection of stromal cells by adeno-cre.

### 3.3. Trp53 Alterations Accelerate Differentiation, Local Invasion, and Metastasis

We observed an increase in locally advanced tumors with increasingly complex genotypes (Figure 1J, *p* = 0.15), which underscores the role of acquired *Kras* and *Trp53* mutations during the adenoma-carcinoma sequence. Interestingly, while AKPfl mice developed highly anaplastic but non-metastatic tumors (Figure 2E), AKPr mice exhibited more differentiated tumors as well as mesenteric lymph node (Figure 2G) and hepatic metastases (Figure 1F and Figure 2H, Table S7. Pulmonary metastases were seen in one AK mouse in the survival analysis group which was euthanized after 262 days (Figure 2I).

### 3.4. Trp53 Loss and Point Mutation Have Differential Effects on Survival and in Vitro Cell Migration

**Figure 4:**
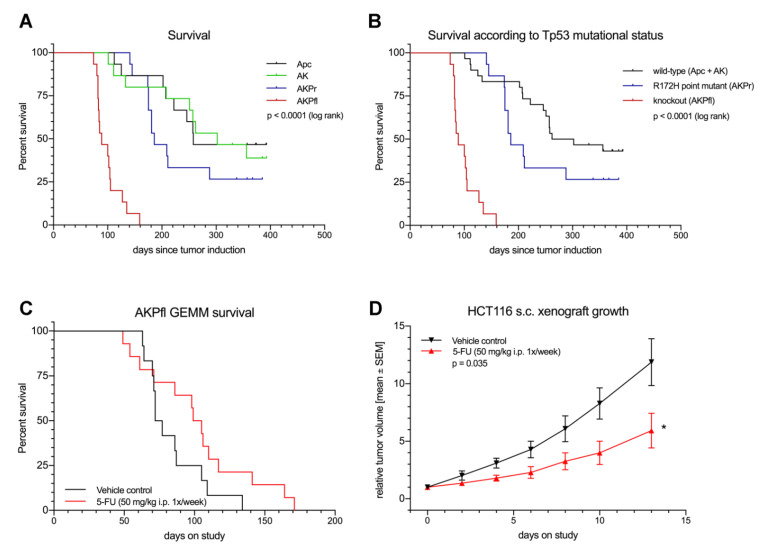
The GEMM mimics the survival and treatment response of human CRC more accurately than a xenograft model. (**A**) Survival of the GEMM depending on the genotype. (**B**) Influence of *TP53* mutational status on survival in the CRC GEMM. (**C**,**D**) Treatment experiments. (**C**) Kaplan–Meyer survival curves of AKPfl mice treated with 5-FU (50 mg/kg i.p the same regimen. * p < 0.05. Apc, Apc^fl/fl^ mice; AK, Apc^fl/fl^/Kras^LSL-G12D/+^ mice; AKPr, Apc^fl/fl^/Kras^LSL-G12D/+^/Trp53^LSL-R172H/+^ mice; AKPfl, Apc^fl/fl^/Kras^LSL-G12D/+^/Trp53^fl /fl^ mice.

While the addition of oncogenic *Kras* to Apc mice (=AK mice) did not influence median survival (258 (Apc) vs. 302 (AK) days, *p* = 0.97), mice with an additional oncogenic mutation of *Trp53* (AKPr mice) had a borderline significant shorter median survival (186 days, *p* = 0.16). Complete knockout of *Trp53* resulted in a strikingly diminished median survival of 89 days (*p* < 0.0001) (Figure 4A). When compared to Trp53 WT mice, the hotspot point mutant of *Trp53* (R172H) did not significantly reduce survival in the GEMM (186 vs. 282 days, *p* = 0.13) (Figure 4B). Conversely, loss of *Trp53* significantly decreased survival (89 vs. 282 days, *p* < 0.0001) (Figure 4B).

In contrast to human CRC patients, who mostly succumb to metastatic disease, bowel obstruction due to local tumor growth, was the most frequent cause of death in the GEMM. In AKPfl mice, distant tumor manifestations rarely occurred and did not influence survival. On the other hand, in the AKPr group, 28.6% of mice developed distant tumor manifestations such as liver metastasis at 168 days after tumor induction. This is in line with data from other groups describing the increased invasive activity of tumor cells harboring the *Trp53* R172H hotspot mutation [23].

In order to confirm the increased migratory activity of AKPr cells, we performed a migration assay of tumor cells derived from AKPfl and AKPr tumors. As expected, the migratory activity of AKPr tumor cells was much higher than in AKPfl cells (Figure S2). In vivo, however, despite the increased metastatic activity of AKPr tumors, the rapid local growth of AKPfl tumors led to earlier death secondary to bowel obstruction.

### 3.5. GEMM Tumors Mimic Human Response to Treatment

We hypothesized that the short survival and rapid growth of AKPfl tumor cells might lead to a higher susceptibility to a cytotoxic agent such as 5-FU. Therefore, we chose AKPfl mice as the model in this experiment.

Similarly to what is observed in patients [30], 5-FU monotherapy showed only moderate activity in the GEMM and did not lead to a statistically significant survival benefit (Figure 4C). For comparison, we conducted the same experiment in a cell line-based xenograft model (Figure 4D). In this experiment, the treated tumors showed a clear reduction in growth rate. The GEMM model thus mimics the human response to 5-FU chemotherapy more realistically than cell line-based mouse models.

### 3.6. Mutations in Trp53 Destabilize the Genetic Integrity of Mouse Tumors

We next analyzed the impact of different *Trp53* alterations on the mutational landscape of the resulting tumors via next-generation exome sequencing.

**Figure 5:**
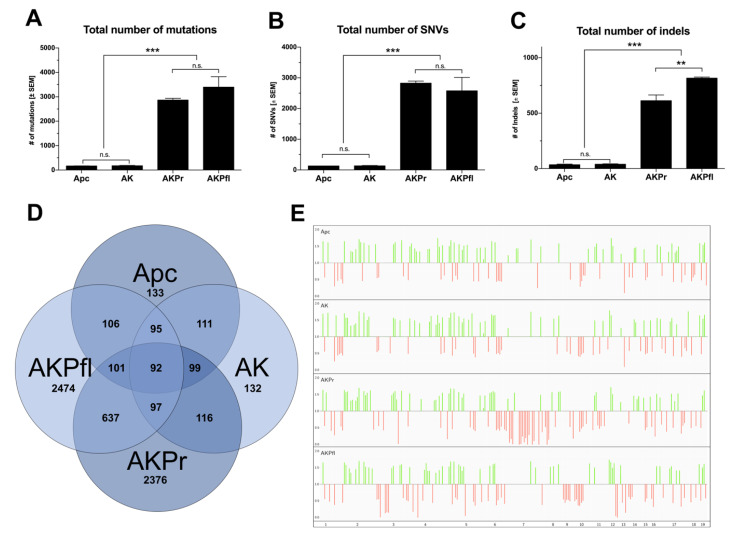
Number of mutations increase considerably in *Trp53*-alterated tumors. (**A**–**C**) Total number of mutations, SNVs and INDELs in tumors of all genotypes. (**D**) Overlapping mutations (TCGA>1%) in all genotypes (Table S3 for list). (**E**) Copy number alterations in all genotypes. Green: gains. Red: losses. ** *p* < 0.01; *** *p* < 0.001; n.s.: not significant. Apc, Apc^fl/fl^ mice; AK, Apc^fl/fl^ / Kras^LSL-G12D/+^ mice; AKPr, Apc^fl/fl^ / Kras^LSL-G12D/+^ / Trp53^LSL-R172H/+^ mice; AKPfl, Apc^fl/fl^ / Kras^LSL-G12D/+^ / Trp53^fl /fl^ mice.

All GEMMs developed numerous new single nucleotide variants (SNVs) over time, demonstrating that the GEMM recapitulates the genetic progression observed in human CRC. As expected, both the average number of mutated genes (Figure 5A–C) and mutations (Figure S3A–C) were significantly higher in *Trp53*-mutant tumors than in Apc and AK mice (Figure 5A–C). To narrow this down to the most relevant mutations, we considered only genetic alterations shared between all mice of each genotype in the subsequent analyses (Figure 5D). Here, again, *Trp53* mutant tumors exhibited a higher number of mutated genes as compared to *Trp53* WT tumors (2430 vs. 132.5, *p* < 0.0001). Knockout of *Trp53* induced the highest number of mutations among all the genotypes. In line with previous data indicating mutual exclusivity of *KRAS* and *BRAF* mutations [31], no *Braf* mutations were seen in any genotype (Table S1). In addition to genotype-exclusive mutations, we also found numerous mutated genes shared among genotypes (Figure 5D and Table S2).

### 3.7. Many Key Mutations Are Shared between Human and Murine CRC

Although, in general, there was only ~5% mutational overlap (4.2–6.1%) between murine and human CRC (Table 1A, Table S1), many important driver mutations known in human CRC spontaneously developed in the murine tumors. Despite relatively stable genomes in Apc and AK mice, the addition of the oncogenic *Kras* G12D mutation consistently induced mutations in *Casc1* (Cancer susceptibility candidate 1), an anti-apoptotic gene. This is in line with previous data from both human [32] and murine [33] lung cancer, in which *CASC1/Casc1* mutations were shown to often coincide with oncogenic *KRAS/Kras*. Both *Trp53* R172H and loss of *Trp53* triggered multiple new oncogenic mutations (Supplementary Table S1) well-known for their role in CRC, including protein-modifying mutations in *Arid1a*, *Arid1b*, *Atm*, *Kmt2a/b/d* (*Mll1/2/4*), *Cdh1*, *Cdk4/6, Erbb2* and *Jak3,* which are among the most frequent oncogenic mutations in human CRC.

### 3.8. Differential Effects of Trp53 Mutation and Knockout on Tumor Biology

A total of 108 (AKPr) and 103 (AKPfl) genes were mutated in the GEMM, which are also present in >1% of the patients in the TCGA cohort. About 60% of these mutations were unique to the respective genotype (Table 1B and Table S3). Despite comparable absolute numbers of mutations, the resulting number of affected pathways shared with the TCGA>1% cohort was significantly higher in AKPr (99) than in AKPfl (46): 33 altered pathways were present in both genotypes, representing 33.33% of pathways in AKPr and 71.74% in AKPfl. Interestingly, AKPr but not AKPfl mice presented mutations in migration and survival pathways (Table 1C and Table 2), which coincides with a strikingly increased migratory activity measured in AKPr vs. AKPfl cells (Figure S2). In addition, immune modulating pathways, such as IL-2 and Dap12, were altered in AKPr which may enable circulating tumor cells to survive in circulation [34]. These findings may at least in part explain the increased metastatic activity in AKPr as compared to AKPfl.

In both AKPr and AKPfl genotypes, several proliferation-associated pathways were enriched, albeit different ones (Table 2), suggesting distinct mechanisms of pathway alteration depending on the type of mutation in *Trp53*.

### 3.9. Copy Number Variation and Pathway Analyses Reveal Similarities between Human and Murine CRC

Chromosomal copy number variation (CNV) analysis revealed no major differences between Apc and AK mice (Figure 5E). However, striking differences between AKPfl and AKPr were noted: while in AKPr, losses on chromosome 7 and gains on 14 and 15 were found, AKPfl exhibited losses on chromosomes 9 and 13 and gains on chromosome 12.

To identify genetic alterations that indirectly affect CRC oncogenic pathways, we performed a pathway analysis using the Reactome pathway database [35,36]. In Apc and AK mice, we did not detect any major alterations in the pathways involved in CRC progression aside from the artificially activated pathways. In AKPr and AKPfl tumors, virtually all major oncogenic pathways are known to play a role in CRC were affected, including chromatin organization and modification, DNA repair, transcription of growth factors and their receptors, cell proliferation and gene expression (Table S4). Comparison between the altered pathways in murine and human CRC using DAVID (the database for annotation, visualization and integrated discovery), a functional annotation tool [37], suggests a similar route to oncogenic progression in mice and humans (Table S5).

### 3.10. Spontaneous Development of Microsatellite Instability in Murine CRC

As several mutations in mismatch-repair (MMR) genes were found in the *Trp53*-altered genotypes, including *Msi2* and *Msh3* (AKPr) and *Msi2*, *Msh4*, *Msh5*, *Msh3* (AKPfl) (Table S1), we aimed to determine the MSI status of the sequenced tumors using a set of three long mononucleotide microsatellites (≥23 repetitive units) as a diagnostic marker panel (Table S6) as described previously [38]. While the majority of tumors proved to be microsatellite-stable (MSS), 2 out of 3 markers were positive in an AKPfl tumor, which was therefore diagnosed as high-grade microsatellite unstable (MSI-H). This tumor also exhibited protein-altering mutations in the MMR genes *Msi2*, *Msh4* and *Msh5*. Overall, these results provide evidence for the spontaneous development of microsatellite instability in the GEMM as well as external validation of the sequencing results.

## 4. Discussion

We here present a novel GEMM of non-hereditary CRC, which is able to recapitulate the adenoma-carcinoma sequence and the multi-hit genetic evolution in colorectal carcinogenesis. We comprehensively characterized the model, demonstrated its genetic and molecular similarities to human CRC as well as its advantages in preclinical trials. Using this GEMM, we investigated the differential effects of various genetic alterations in the *Trp53* gene in CTC.

There is a number of GEMMs for CRC known, most notably the Apc^min^ mouse [39]. Since these animals develop multiple tumors throughout the small and large intestine and die of the benign tumor load before invasive tumors can develop, other approaches have been undertaken in order to confine the tumor formation to smaller regions of the colon. One such attempt is using inducible, colon-specific cre alleles such as Car1-Cre^ERT2^ mice [40]. Although the tumors in this model are confined to the proximal colon, there are still >10 neoplastic foci/square millimeter of colonic mucosa.

In human CRC, the initial and rate-limiting mutation is the inactivation of *APC* leading to adenoma formation [2]. Similar to the human disease, in GEMM this initial step is followed by multiple new mutations, recapitulating the genetic progression of CRC. In addition, the GEMM tumors exhibit similar biological and clinical changes upon mutations in *Kras* and *Trp53* in comparison to human CRC, including genomic destabilization, faster progression, de-differentiation and increased metastatic activity.

The long latency of adenocarcinoma formation in Apc and AK mice suggests the requirement of spontaneous acquisition of additional mutations in *Trp53* or other genes. It underlines the role of *Trp53* as a gatekeeper for malignant tumor formation, which is again well in line with human CRC.

The tumors with complete loss of p53 expression (AKPfl) exhibit more vigorous growth, leading to early local complications such as bowel obstruction. In contrast, tumors with point mutations in the *Trp53* gene proliferate slower but exhibit increased metastatic capacity. In line with previous investigations on *Trp53* mutations [23,41], this indicates not only incomplete dominant negative effects of the mutant P53 protein and some retained function of the WT allele but also functional gains of *Trp53* R172H, increasing the metastatic activity of tumor cells with this mutation. Exome sequencing of AKPr revealed numerous mutations and pathways involved in migration and invasion of cancer cells as well as immune modulating pathways, which may increase the metastatic activity as well as the chance of tumor cell survival during the process of metastasis [34].

A pivotal requirement of a GEMM is its predictive value in drug trials. As we aimed to demonstrate the predictive superiority of the GEMM over a classical xenograft model, we treated the mice with weekly 5-FU boluses, a regimen which has proven effective in xenograft models [42,43] but failed in patients [44]. Similar to the human situation, 5-FU showed no activity in the GEMM while significant growth retardation was seen in a subcutaneous xenograft model, indicating a more realistic response prediction in the here presented GEMM.

While the data presented here is derived from mice with four different genotypes, the model can be easily adapted for multiple other CRC subtypes by introducing other conditional mutations such as *Pik3ca*, *Pten,* or *Smad4*. The model may, therefore, serve as a platform to test therapeutic interventions in different genetic entities of CRC. This has been shown before in murine *Braf* V600E CRC [45] and maybe a step forward towards preclinical modeling of personalized oncology.

## 5. Conclusions

In conclusion, the GEMM described here closely mimics human CRC both clinically and molecularly. It enables both molecular studies on tumor genetics as well as preclinical therapeutic studies, which makes it an ideal model for basic molecular oncology and drug development in CRC.

## Figures and Tables

**Table 1:** A. Comparison of Mutations in the GEMM and The Cancer Genomic Atlas (TCGA) Dataset. “TCGA >1%” Refers to Mutations Present in >1% of Human CRC Tumors. B and C. Overview of Shared and Unique TCGA Mutations (B) and Resulting Affected Pathway (C) between AKPr and AKPfl Genotypes.

**A**					
**Genotype**	**Total**	**TCGA**	**% TCGA**	**TCGA >1%**	**% TCGA >1%**
Apc	133	8	6.0	6	4.5
AK	132	10	7.6	8	6.1
AKPr	2381	123	5.2	108	4.5
AKPfl	2479	127	5.1	103	4.2
**B**					
**Genotype**	**TCGA>1%** **Mutations**	**Shared**	**Unique**	**% Unique**	**% Overlap**
AKPr	108	41	67	62.04	37.96
AKPfl	103	41	62	61.19	39.81
**C**					
**Genotype**	**Affected pathways**	**Shared**	**Unique**	**% Unique**	**% Overlap**
AKPr	99	33	66	66.67	33.33
AKPfl	46	33	13	28.26	71.74

**Table 2:** Major Altered Pathways in AKPr and AKPfl Mice.

Pathways	AKPfl	AKPr	Function
Adhesion	X	-	Adhesion
Hedgehog	X	-	Differentiation
DNA Repair *	X	X	DNA maintenance
Methylation	-	X	DNA methylation
Chromatin regulation	-	X	Gene expression
Nuclear transcription	X	X	Gene expression
Regulation of immune response	X	X	Immune modulation
Dap12	-	X	Immune modulation, tumor cell survival
IL-2	-	X	Immune modulation, identifying self and non self
Angiopoeitin	-	X	Migration, survival
IL-3, -5, GM-CSF	-	X	Migration, survival
Cell cycle regulation	X	-	Proliferation
Egfr *	X	X	Proliferation
Erbb	X	X	Proliferation
Fgfr1	X	X	Proliferation
Igfr	-	X	Proliferation
Igfr1 *	X	X	Proliferation
IL-6	X	-	Proliferation
Insulin pathway *	X	X	Proliferation
Proto-oncogene	-	X	Proliferation
Ptk6 *	X	X	Proliferation
Raf	-	X	Proliferation
Ras *	X	X	Proliferation
Ras family of oncogenes	X	X	Proliferation
Erk *	X	X	Proliferation, migration
Growth factor /receptor transcription	X	X	Proliferation, migration
Interleukins	X	X	Proliferation, migration
Mapk	X	X	Proliferation, migration
Notch *	-	X	Proliferation, migration
Notch *	X	-	Proliferation, migration
p38	X	X	Proliferation, migration
Vegfr2	-	X	Proliferation, migration
Scf-Kit	X	X	Proliferation, survival

* Pathways activated by different protein interactions in AKPfl and AKPr, respectively.

## Data Availability

NGS datasets have been uploaded to the Gene Expression Omnibus (GEO) database (https://www.ncbi.nlm.nih.gov/geo/).

## Supplementary Materials

The following are available online at https://www.mdpi.com/2072-6694/13/4/808/s1, Table S1: List of genes which are mutated in both the GEMM and the The Cancer Genome Atlas (TCGA) human CRC dataset, Table S2: List of mutated genes shared between different genotypes, Table S3: List of unique and common TCGA >1% genes between AKPr and AKPfl tumors, Table S4: Overview of all the enriched pathways between AKPfl and AKPr, Table S5: Comparison of enriched pathways between human and mouse for AKPfl and AKPr genotypes, Table S6: Mutations in mismatch repair genes and MSI analysis, Table S7: Overview of the metastatic pattern seen in the different genotypes, Figure S1: Poorly differentiated and anaplastic tumors are more frequent in *Trp53*-mutant GEMMs, Figure S2: Tumor cells derived from AKPr mice have higher migration ability in comparison with tumor cells derived from AKPfl mice, Figure S3. The GEMMs recapitulate the increased mutation number observed in *TP53*-mutant human CRC.
