# Supplementary material for: Differential Effects of Trp53 Alterations in Murine Colorectal Cancer
Source: Cancers (Basel). 2021 Feb 15;13(4):808. doi: 10.3390/cancers13040808 (PMC7919037; doi:10.3390/cancers13040808)
Supplement: Supplementary file 1 [file cancers-13-00808-s001.zip › cancers-1097481-Supplementary Materials/cancers-1097481-Supplementary Materials.pdf]

cancers

MDPI

Supplementary Materials: Differential Effects of Trp53 Alterations in Murine Colorectal Cancer

Alexander M. Betzler Lahiri K. Nanduri Barbara Hissa, Linda Blickensdörfer, Michael H. Muders, Janine Roy, Moritz Jesinghaus, Katja Steiger, Wilko Weichert, Matthias Kloor, Barbara Klink, Michael Schroeder, Massimiliano Mazzone, Jürgen Weitz, Christoph Reissfelder, Nuh N. Rahbari and Sebastian Schölch

Citation: Betzler, M.A.; Nanduri, K.L.; Hissa, B.; Blickensd, L.; Muders, H.M.; Roy, J.; Jesinghaus, M.; Steiger, K.; Weichert, W.; Kloor, M.; et al. Differential Effects of Trp53 Alterations in Murine Colorectal Cancer. *Cancers* **2021**, *13*, 808. https://doi.org/10.3390/cancers13040808

Academic Editor: Masako Nakanishi

Received: 18 January 2021

Accepted: 8 February 2021

Published: 15 February 2021

Publisher's Note: MDPI stays neutral with regard to jurisdictional claims in published maps and institutional affiliations.

Copyright: © 2021 by the authors. Licensee MDPI, Basel, Switzerland. This article is an open access article distributed under the terms and conditions of the Creative Commons Attribution (CC BY) license (http://creativecommons.org/licenses/by/4.0/).

1. Supplementary Methods

1.1. Animal experiments

Tumor induction via segmental adeno-cre infection: The procedure to achieve segmental adeno-cre infection in the colon has been described in detail before [20] and has been adapted from a previously published model [19,46]. Briefly, mice were anesthetized by sevoflurane inhalation (2.5–3.0 Vol%) and subcutaneous (s.c.) injection of buprenorphine. A lower midline laparotomy was performed and the distal colon identified and clamped with a Yasargil vascular clamp. Then a flexible Teflon tube was inserted transanally and the distal colon flushed with saline 0.9% to remove feces. Then a second clamp was placed ca. 5 mm distal of the first clamp and the isolated segment was inflated with trypsin solution (1%). After 10 minutes of incubation, the trypsin was aspirated, followed by another saline flush. Then the segment was inflated with highly concentrated adenovirus solution (1×10^{11} PFU/ mL) and incubated for 30 minutes. Next, the tube and clamps were removed and the abdomen closed with running PDS 5-0 sutures and surgical wound clips. Pivotal differences between our protocol and the previously published protocol [19] are: 1) Flushing prior to trypsin incubation makes overnight fasting dispensable, which drastically reduces the stress on the animals and 2) the higher viral concentration used in our protocol makes mechanical scrubbing of the bowel wall dispensable, reducing the rate of perforation and postoperative mortality.

Mouse colonoscopy: To assess the growth of colon tumors, the mice were subjected to 2-weekly colonoscopy with the Mouse ColoView System Mainz (Karl Storz, Tuttlingen, Germany). Mice were anesthetized via sevoflurane inhalation; the distal colon was flushed with NaCl via a soft teflon tube and sigmoidoscopy was performed.

Survival study: Tumors were induced and mice were allocated to the groups according to their genotype. Animal health was evaluated daily. Whenever signs of distress were identified, mice were euthanized a death event was recorded.

Treatment experiments: Tumors were induced via segmental adeno-cre infection in AKPfl mice as described previously. Colonoscopy was subsequently performed every other week. Once tumor growth was identified, mice were randomized to the treatment cohorts and treatment was initiated. The treatment protocol consisted in giving, once a week, intraperitoneal injections (i.p.) containing 50 mg/kg Fluorouracil (5-FU) diluted in 100 µL NaCl 0.9%. Normal saline was also used as vehicle control and administered in the same volume and frequency as 5-FU.

HCT116 s.c. experiment: NOD.Cg-Prkdc^{scid} Il2rg^{tm1Wjl}/SzJ (NOD scid gamma; NSG) mice were purchased from Charles River (Sulzfeld, Germany). 1.0×10^6 HCT116 cells were injected subcutaneously into both flanks of donor animals. Once the resulting tumors reached a size of 10–12 mm in largest diameter, the tumors were excised and 1×1 mm fragments were implanted subcutaneously into both flanks of NSG mice. Tumor volumes were measured every other day with a caliper. The formula: $V = \frac{d\ x\ d\ x\ D}{2}$, in which d is the minor tumor axis and D is the major tumor axis, was used to estimate the tumor volume. Treatment (5-FU 50 mg/kg i.p. qw or NaCl 0.9% i.p. qw as vehicle control) was initiated when tumors reached a size of 6 mm in largest diameter. Mice were euthanized when tumors reached 20 mm in largest diameter or suffering of the animals became apparent.

1.2. Histology

The FFPE tissue specimens were sectioned and stained with hematoxylin / eosin (H/E) by standard laboratory methods. Sarcomas were excluded from all further analyses. Histologic diagnoses and terminology were performed according to the consensus guidelines on rodent intestinal neoplasia [25,26].

Grading ranged from well differentiated (G1) to moderately (G2), poorly differentiated (G3) and undifferentiated (anaplastic, for clarity reasons here named G4) tumors. Only invasive adenocarcinomas were subjected to pathological grading.

T staging was done similar to human T staging of colorectal tumors and according to the 8th edition of the UICC TNM classification of malignant tumors (www.uicc.org). The classification ranges from T1 (invasion into submucosa) to T2 (invasion into muscularis propria), T3 (invasion through the muscularis propria into pericolorectal tissues) and T4 (penetration of the visceral peritoneum).

1.3. Immunohistochemistry

The slides were deparaffinized according to standard protocols, pretreated with citrate buffer pH6 (Epitopal Retrival Solution 1, ER1), stained with CDX2 (EPR2764Y, Abcam, Berlin, Germany), EpCAM (ab71916, Abcam, Berlin, Germany), Beta-Catenin (E247, Abcam, Headquarters) and P53 (CM5, Leica, Wetzlar, Germany) and visualized with the BOND polymer refine detection system (Leica, Wetzlar, Germany) with the anti-rabbit HRP labeled secondary antibody (Leica, Wetzlar, Germany) polymer.

1.4. Microsatellite Instability Testing

Tumors were analyzed for microsatellite instability (MSI) using a microsatellite panel of three long mononucleotide repeat stretches highly sensitive for the detection of MSI [38]. DNA fragment analysis was performed as described previously [38]. The DNA fragments were amplified using fluorescently labeled oligonucleotide primers and visualized using an ABI3130xl sequencer (Applied Biosystems, Darmstadt, Germany) [38]. Markers were scored as unstable if novel peaks occurred in tumor compared to normal tissue. Tumors were scored as MSI if at least two of the 3 analyzed markers showed instability.

1.5. Next Generation Sequencing

DNA was isolated from frozen specimens of tumor and corresponding peripheral blood mononuclear cells (PBMC) using the QiAMP Blood mini and QiAMP micro kits (Quiagen, Düsseldorf, Germany). Paired-end Enriched Exome Sequencing (Agilent SureSelect Mouse All Exon, read length 100 bp) was performed on an Illumina HiSeq 2000 in the Genomics and Proteomics Core Facility of German Cancer Research Center (DKFZ) Heidelberg. Alignment and mapping were performed using DNA Nexus Technology. DNA from healthy littermates was used as wild type controls.

1.6. Next-Generation Sequencing (NGS) Data Analysis

We aligned the resulting reads from above to the Grcm38 reference genome with BWA [47], applied GATK [48] base quality score recalibration, INDEL realignment, duplicate removal, and performed SNP and INDEL discovery. We then applied standard hard filtering parameters or variant quality score recalibration as recommended by GATK best practices [49,50]. Variants from the control were subtracted to exclude background SNPs. Variants were then compared to variants in the TCGA CRC dataset [1] accessible via cBioPortal (www.cbioportal.org) [51,52].

Pathway analysis: The pathway analysis was performed using the CRC-relevant mutated genes as described above. For the comparative pathway analysis between murine and human genomes the DAVID functional annotation tool [37] was used. The murine gene identifiers were converted to the equivalent human identifiers using DAVID and later used to identify the enriched human pathways. A DAVID enrichment score >2 and

a FDR (false detection rate) ≤0.1 was used as a cutoff for consideration. For a detailed pathway analysis of the mouse genotypes in comparison to human, Reactome, a pathway analysis tool [35,36,53] with a feature project for humans, was used with an FDR of ≤0.1. All genes which were mutated both in the GEMM and the TCGA cohort were analyzed. Here too, the murine gene identifiers were converted to human gene identifiers and the relevant enriched pathways were listed.

1.7. Copy Number Variant Analysis

For CNV analysis, mapping was performed using the bwa-mem (V0.7.8) algorithm to the reference mouse assembly, GRCm38, downloaded from the mouse genomes project of the Sanger Institute. Copy Number Variation was later called using R (V3.3.2) and ExomeDepth package (V1.1.10), which fits a hidden Markov model to the read depth data to call copy number variations. The required bed file for the analysis was downloaded from UCSC Gene Browser. Copy number variations were called significant after comparing to the sequence of the reference mouse genome (C57Bl/6N and exome seq data of a wild type littermate of the GEMM) and a transition probability of 10^{-4}.

1.8. Migration assay

Primary tumor cells from AKPr and AKPfl tumors were collected, cut into small pieces, digested for 1 hour in Collagenase-dispase enzyme solution (Sigma Aldrich, Taufkirchen, Germany) and centrifuged at 200g for 3 minutes. The pellet was washed with PBS, filtered through a 100 µM filter mesh and centrifuged again at 200g. The resulting organoid pellet was resuspended in Matrigel (Corning, Kaiserslautern, Germany) and 50 µl organoid solution/ well was plated in a pre-warmed 48 well plate. 200 µl of growth medium (DMEM, 1x Pen/Strep, 50 nM Hepes, 50 ng/ml EGF, 50% Wnt Conditioned medium (CM), 10% Rspondin, 10% Noggin, 1x B27 and 1x N2 and 1x Primocin) was added. Later, the organoids were submitted to serum-starvation for 36 hours before the beginning of the experiment. The cells were then trypsinized, washed once with 1x PBS (GIBCO, Dreireich, Germany) and stained with CellTracker® green (Molecular Probes, Dreireich, Germany) for 30 minutes according to the manufacturer's protocol. 1.0 x 10^4 cells were seeded, in triplicates, on a 24-well plate containing 8 µm mesh size transwells (Corning, NY,USA). The upper chamber was filled with basal medium (DMEM/F12 supplemented with 1x penicillin/streptomycin, 1x Glutamax and 100nM HEPES buffer) whereas the lower chamber was filled with growth medium (basal medium supplemented with 1x B27, 1x N2, 1x primocin and 1.25 mM N-acetylcysteine). The cells were seeded in triplicates per condition. After 72 hours, the upper chamber was lifted and the cells on the top of the transwells were carefully removed using a cotton swab. The cells that migrated to the bottom side of the mesh were then fixed in 5% glutaraldehyde, stained with 1% DAPI in methanol and counted under an epifluorescent microscope (Leica, Wetzlar, Germany). Three fields per condition were counted under a 10x objective.

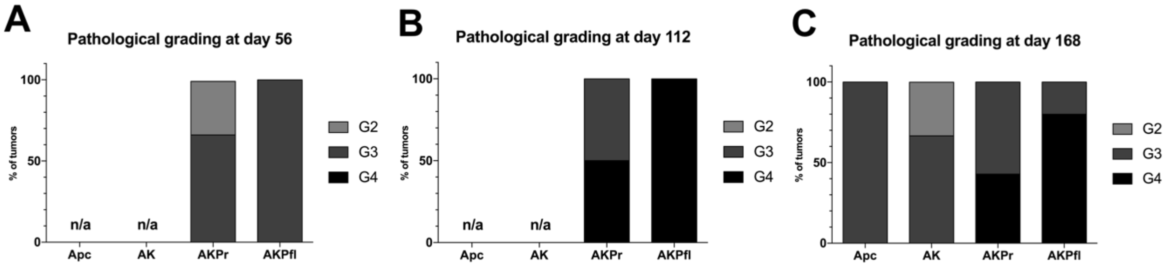

Figure S1. Poorly differentiated and anaplastic tumors are more frequent in *Trp53*-mutant GEMMs. Pathological grading at 56 (**A**), 112 (**B**) and 168 days (**C**) after tumor induction. At 56 and 112 days after tumor induction no macroinvasive tumors were seen in Apc and AK mice. Apc, $Apc^{fl/fl}$ mice; AK, $Apc^{fl/fl}$ / $Kras^{LSL\text{-}G12D/+}$ mice; AKPr, $Apc^{fl/fl}$ / $Kras^{LSL\text{-}G12D/+}$ / $Tp53^{LSL\text{-}R172H/+}$ mice; AKPfl, $Apc^{fl/fl}$ / $Kras^{LSL\text{-}G12D/+}$ / $Tp53^{fl/fl}$ mice.

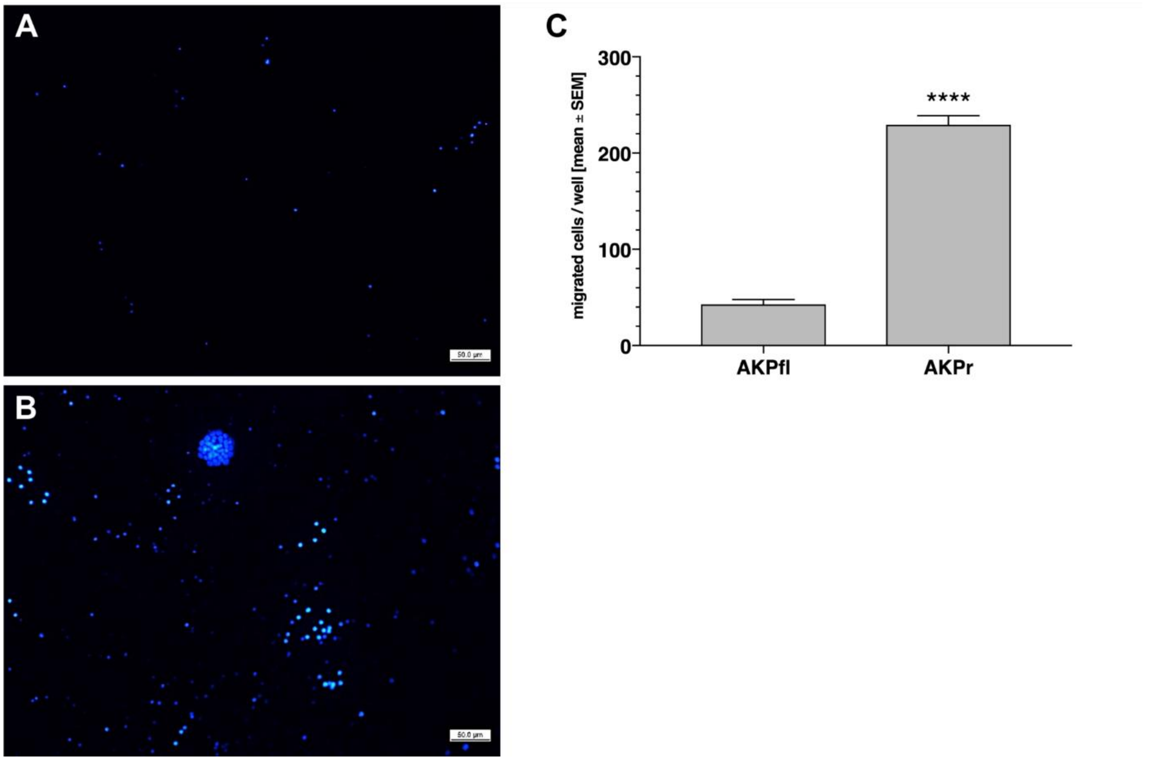

Figure S2. Tumor cells derived from AKPr mice have higher migration ability in comparison with tumor cells derived from AKPfl mice. Migration assay of AKPfl and AKPr cells. A + B. DAPI staining of migrated AKPfl (A) and AKPr (B) cells. C. Quantification of migrated cells. AKPr, Apc$^{fl/fl}$ / Kras$^{LSL\text{-}G12D/+}$ / Tp53$^{LSL\text{-}R172H/+}$ mice; AKPfl, Apc$^{fl/fl}$ / Kras$^{LSL\text{-}G12D/+}$ / Tp53$^{fl/fl}$ mice. **** $p < 0.0001$.

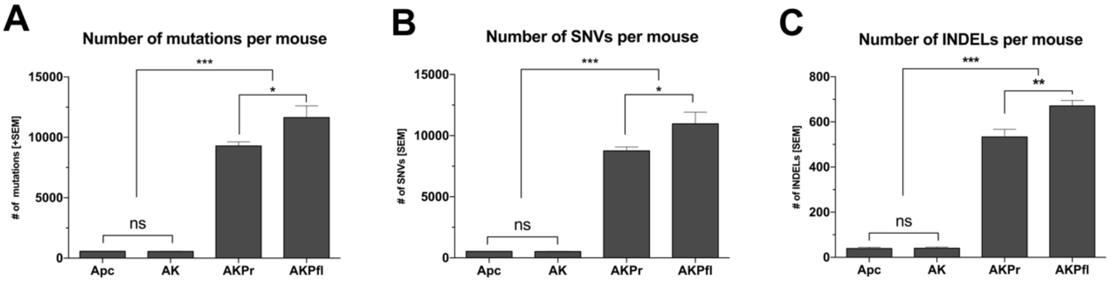

Figure S3. The GEMMs recapitulate the increased mutation number observed in *TP53*-mutant human CRC. Overview of the total number of mutations **(A)**, SNVs **(B)** and INDELs **(C)** in tumors of all genotypes (for the number of mutated genes, see Figure 6 A–C). * $p < 0.05$; ** $p < 0.01$; *** $p < 0.001$. Apc, Apc$^{fl/fl}$ mice; AK, Apc$^{fl/fl}$ / Kras$^{LSL\text{-}G12D/+}$ mice; AKPr, Apc$^{fl/fl}$ / Kras$^{LSL\text{-}G12D/+}$ / Tp53$^{LSL\text{-}R172H/+}$ mice; AKPfl, Apc$^{fl/fl}$ / Kras$^{LSL\text{-}G12D/+}$ / Tp53$^{fl/fl}$ mice.
